# Cerebrospinal fluid biochemical studies in patients with Parkinson's disease: toward a potential search for biomarkers for this disease

**DOI:** 10.3389/fncel.2014.00369

**Published:** 2014-11-11

**Authors:** Félix J. Jiménez-Jiménez, Hortensia Alonso-Navarro, Elena García-Martín, José A. G. Agúndez

**Affiliations:** ^1^Section of Neurology, Hospital Universitario del SuresteMadrid, Spain; ^2^Department of Biochemistry and Molecular Biology, University of ExtremaduraCáceres, Spain; ^3^AMGenomicsCáceres, Spain; ^4^Department of Pharmacology, University of ExtremaduraCáceres, Spain

**Keywords:** Parkinson's disease, cerebrospinal fluid, biological markers, neurotransmitters, oxidative stress, tau protein, alpha-synuclein, beta-amyloid

## Abstract

The blood-brain barrier supplies brain tissues with nutrients and filters certain compounds from the brain back to the bloodstream. In several neurodegenerative diseases, including Parkinson's disease (PD), there are disruptions of the blood-brain barrier. Cerebrospinal fluid (CSF) has been widely investigated in PD and in other parkinsonian syndromes with the aim of establishing useful biomarkers for an accurate differential diagnosis among these syndromes. This review article summarizes the studies reported on CSF levels of many potential biomarkers of PD. The most consistent findings are: (a) the possible role of CSF urate on the progression of the disease; (b) the possible relations of CSF total *tau* and phospho*tau* protein with the progression of PD and with the preservation of cognitive function in PD patients; (c) the possible value of CSF beta-amyloid 1-42 as a useful marker of further cognitive decline in PD patients, and (d) the potential usefulness of CSF neurofilament (NFL) protein levels in the differential diagnosis between PD and other parkinsonian syndromes. Future multicentric, longitudinal, prospective studies with long-term follow-up and neuropathological confirmation would be useful in establishing appropriate biomarkers for PD.

## Introduction

The diagnosis of Parkinson's disease (PD) in live patients is fundamentally clinical, and is based on the presence of its cardinal signs (rest tremor, rigidity, bradykinesia, and postural instability), and the absence of atypical data for idiopathic PD. The final confirmation of the diagnosis is made by post-mortem neuropathological analysis. To date, there are no definitive biomarkers to make an accurate differential diagnosis with other parkinsonian syndromes.

Because the cerebrospinal fluid (CSF) is in close contact with the extracellular space of the brain, it is believed that many of the biochemical modifications in the brain should be reflected in the CSF. Therefore, CSF has been widely investigated in PD and in other parkinsonian syndromes with the aim of acquiring knowledge on the pathogenesis of this disease. This article summarizes the data on analyses performed in the CSF of patients diagnosed with PD compared with controls, with regard to: (1) concentrations of neurotransmitters (mainly monoamines and their metabolites), neuromodulators, and related substances as possible biological markers of the disease itself or its complications; (2) concentrations of endogenous neurotoxins; (3) status of oxidative stress markers or substances which could be related with the induction of oxidative stress or with “neuroprotection” against it; (4) status of inflammation and immunological markers, neurotrophic and growth factors, and (5) concentrations of proteins related with the pathogenesis of PD or other compounds.

The aim of this review is to provide an extensive descriptive overview of studies published on this issue (including references to many reports in the last six decades which have historical interest).

## Search strategy

References for this review were identified by searching in PubMed from 1966 until June 20, 2014. The term “*Parkinson's disease*” was crossed with “*cerebrospinal fluid*” and “*blood brain barrier*,” and the related references were selected. Table 1 summarizes a classification of the diverse types of compounds which have been analyzed in the CSF of PD patients in accordance with the search.

**Table 1:** **Relation and classification of compounds measured in CSF of PD**.

(A) Neurotransmitters, neuromodulators, and related substances
Dopamine (DA) metabolites: dihydroxyphenylacetic acid (DOPAC) and homovanillic acid (HVA), 3-orthomethylDOPA (3-OMD) Serotonin (5-hydroxytryptamine or 5-HT) metabolites or precursors: 5-hydroxytryptophan (5-HTP), 5-hydroxyindoleacetic acid (5-HIAA), kynurenine, 3-hydroxykynurenine Noradrenalin (norepinephrine or NE) metabolites or precursors: 3-methoxy-4-hydroxy-phenylethylenglycol (MHPG), dopamine-beta-hydroxylase (DBH) Acetylcholine (Ach) and related substances: choline, acetylcholine-esterase (AchE), butiryl-cholin-esterase (BchE) Neurotransmitter amino acids: gamma-amino butyric acid (GABA), glutamate, aspartate, glycine Neuropeptides: substantia P (SP), cholecystokinin-8 (CCK-8), met-enkephalin (MET-ENK), leu-enkephalin (LEU-ENK), dynorphin A(1-8), somatostatin, neuropeptide Y (NPY), beta-endorphin, arginine-vasopressine (AVP), vasoactive intestinal peptide (VIP), delta sleep-inducing peptide (DSIP), alpha-melanocyte-stimulating hormone-like, diazepam-binding inhibitor, neurokinin A, corticotropin-releasing hormone (CRH), adrenocorticotropin hormone (ACTH), beta-lipotropine, angiotensin, chromogranins A and B, secretogranin II, orexin-A/hypocretin-1 Other neurotransmitters: endogenous cannabinoids, β-phenylethylamine Cyclic nucleotides: cyclic adenosine 3′5′ monophosphate (cAMP), cyclic guanosine 3′5′ monophosphate (cGMP) Biopterin derivatives and other cofactors
(B) Endogenous neurotoxins
Tetrahydroisoquinolin (TIQ) derivatives: 2-methyl-6,7-dihydroxy1,2,3,4-TIQ (2-MDTIQ), 1-MDTIQ (salsolinol). 1-benzyl-1,2,3,4-TIQ β-carbolinium cations (BC+s)
(C) Oxidative stress markers
Lipid peroxidation markers: Malonyl-dialdehyde (MDA) (E)-4-hydroxynonenal (HNE) Low density lipoprotein (LDL) oxidation products Schiff bases, conjugated dienes, oxidized proteins, and aldehyde polymers DNA oxidation markers: 8′-hydroxy-2′deoxyguanine (8-OHdG) 8-hydrosyguanosine (8-OHG) 8-OHdG/8-OHG ratio Transition metals and related proteins: iron, ferritin, transferring, copper, cerulopasmin, ferroxidase, manganese, zinc Other metals: selenium, chromium, magnesium, calcium, aluminum, silicon, cobalt, tin, lead, barium, bismuth, cadmium, mercury, molibdenum, nichel, antimony, strontium, thallium, vanadium, wolfram, and zirconium
(D) Inflamatory and immunological markers
Inteleukins (IL) Tumor necrosis alpha (TNF-α) Other: leukotrienes. α-1-antichymotrypsin
(E) Growth and neurotrophic factors
Brain-derived neurotrophic factor (BDNF) Transforming Growth Factors: TGF-α, TGF-β1, TGF-β2 Insulin-like growth factor-1 (IGF-1) and IGF-binding proteins (IGFBPs) Neuroregulins (Epidermal Growth Factor or EGF family)
(F) Proteins involved in the pathogenesis of PD
Microtubular-Associated Protein *Tau* (MAPT) Alpha-synuclein Amiloyd beta Neurofilament proteins Other proteins: DJ-1, UCH-L1
(G) Other compounds

## Neurotransmitters, neuromodulators, and related substances

### Dopamine metabolites

Because the main neurochemical finding in PD is the depletion of dopamine (DA) in the nigroestriatal system (Benito-León et al., 2008), it is to be expected that the CSF concentrations of the main metabolites of DA, dihydroxyphenyl-acetyc acid (DOPAC) and homovanillic acid (HVA), should be decreased. Indeed, many classical studies have shown variable degrees of decrease in the CSF HVA levels of PD patients compared with controls (Bernheimer et al., 1966; Guldberg et al., 1967; Johansson and Roos, 1967; Olsson and Roos, 1968; Gottfries et al., 1969; Curzon et al., 1970; van Woert and Bowers, 1970; Godwin-Austen et al., 1971; Mones et al., 1972; Papeschi et al., 1972; Pullar et al., 1972; Cox et al., 1973; Voto Bernales et al., 1973; Weiner and Klawans, 1973; Granerus et al., 1974; Davidson et al., 1977; Tabaddor et al., 1978; Lovenberg et al., 1979; Cunha et al., 1983; Mann et al., 1983; Cramer et al., 1984; Mena et al., 1984; Pezzoli et al., 1984; Burns et al., 1985; Gibson et al., 1985; Jolkkonen et al., 1986; Liu, 1989; Hartikainen et al., 1992; Strittmatter and Cramer, 1992; Chia et al., 1993; Mashige et al., 1994; Eldrup et al., 1995; Cheng et al., 1996; Strittmatter et al., 1996; Kanemaru et al., 1998; Goldstein et al., 2008). Engelborghs et al. (2003) reported normal CSF DA and HVA, and decreased DOPAC levels. González-Quevedo et al. (1993) described normal CSF HVA levels, Espino et al. (1994) found decreased HVA only in advanced but not in early PD, Parkinson Study Group DATATOP Investigators found normal levels in early PD (LeWitt et al., 2011). Zubenko et al. (1986) described a non-significant trend toward decreased CSF HVA levels in demented PD patients compared with controls. Tohgi et al. (1993a) found correlation of CSF DA and HVA levels with akinesia and freezing of gait.

Although levodopa treatment usually increases CSF HVA levels according to the majority of studies, this is not related with clinical improvement, with some exceptions (Durso et al., 1989), and pre-treatment CSF HVA levels does not predict levodopa response (Weiner et al., 1969; Chase, 1970; Curzon et al., 1970; Bertler et al., 1971; Casati et al., 1973; Cox et al., 1973; Mones, 1973; Weiner and Klawans, 1973; Granerus et al., 1974; Davidson et al., 1977; Liu, 1989; Nishi et al., 1989; Strittmatter et al., 1996; Antkiewicz-Michaluk et al., 1997; Durso et al., 1997; Krygowska-Wajs et al., 1997), except in one study which described an association between relatively high pre-treatment CSF HVA levels and a better response to levodopa (Gumpert et al., 1973). One study failed to show changes in ventricular CSF HVA levels after a single acute administration of levodopa (Moussa et al., 1992). On the other hand, dopamine agonists such as piribedil and bromocriptine decreased significantly both the basal level (McLellan et al., 1975; Rinne et al., 1977) and probenecid-induced accumulations of HVA in CSF (Rinne et al., 1975, 1977), indicating that the drugs reduced the turnover of endogenous dopamine. Amantadine did not change HVA levels (Cox et al., 1973). Tetrahydrobiopterin (Dissing et al., 1989) and L-threo-3,4-dihydroxyphenylserine (precursor or noraderenalin or norepinephrine –NE) (Maruyama et al., 1994) increased CSF HVA levels in PD patients, but to a lesser extent than levodopa.

Friedman et al. (Friedman, 1985) reported an HVA/5-HIAA ratio in PD patients who developed levodopa-induced dyskinesias (LID) which was significantly higher than in PD patients under levodopa therapy and in controls, but Lunardi et al. (2009) found similar HVA/DA ratios in patients with and without LID. CSF DA, levodopa, and HVA levels were similar in PD patients treated with levodopa with wearing-off motor fluctuations to those without this complication of levodopa therapy, while CSF 3-ortho-methyldopa (3-OMD) levels were higher in the fluctuating patients (Tohgi et al., 1991a). CSF DOPAC and HVA were similar in PD patients with and without depression (Kuhn et al., 1996a), and in patients with major depression with PD than in those without PD (Pålhagen et al., 2010). CSF HVA levels were correlated with striatal uptake in PD patients measured with PET imaging with carbon-11-labeled 2β-carbomethoxy-3β-(4-fluorophenyl)-tropane (^11^C-FT) (Ishibashi et al., 2010).

Tohgi et al. (1991b, 1997) found a significant increase in tyrosine, and a significant decrease in CSF levodopa, DA, and 3-OMD in PD patients, which was related with levodopa dosage, and described an additional decrease in 3-OMD in subjects treated with tolcapone (Tohgi et al., 1995a). Other authors reported increased CSF 3-OMD related with levodopa therapy (Antkiewicz-Michaluk et al., 1997; Krygowska-Wajs et al., 1997). On the other hand, Chia et al. (1993) found normal CSF 3-OMD concentrations. Moser et al. (1996) described increased CSF levodopa/3-OMD ratio in PD patients with hallucinations. Iacono et al. (1997) found similar HVA levels in PD patients with postural instability and gait disorders to PD patients without these symptoms.

Although many of the studies of DA metabolites were performed on patients with different types of parkinsonism, with different degrees of severity, and the fact that many of these studies were made using small sample sizes, there is a general consensus that CSF HVA levels are decreased in untreated PD patients and rise after levodopa therapy starts (decreased HVA may not be present in early stages of PD). It is to be expected that low CSF HVA levels should be a reflection of DA depletion in the nigroestriatal system. However, CSF DA metabolite levels are not useful to distinguish between different parkinsonian syndromes and could be normal in early stages of the disease. To our knowledge, no studies have been published regarding the correlation of CSF DA metabolite levels and brain DA levels, although the observation of a correlation between CSF HVA levels and striatal uptake of DA markers in PET imaging (Ishibashi et al., 2010), suggests this correlation.

### Serotonin (5-hydroxytryptamine or 5-HT) metabolites

Several studies have described neuronal loss, and presence of Lewy body in serotonergic raphe nuclei in PD patients (Benito-León et al., 2008). Tohgi et al. (1993b,c, 1997) reported a 15–20% reduction of CSF 5-HT, tryptophan (precursor of 5-HT), kynurenine and 3-hydroxykynurenine (metabolites of tryptophan) levels in PD patients. CSF 5-HT levels showed a negative correlation with the severity of bradykinesia, rigidity and freezing of the gait, and decreased after levodopa therapy. This group also found a correlation between CSF 5-HIAA levels and akinesia and freezing of gait (Tohgi et al., 1993a). In contrast, Engelborghs et al. (2003) described increased 5-HT levels. LeWitt et al. (2013) described increased CSF 3-hydroxykynurenine levels, and Widner et al. (2002) described an increased CSF kynurenine/tryptophan ratio in PD patients.

Several studies have shown reduced CSF levels of 5-hydroxyindoleacetic acid (5-HIAA), the main metabolite of 5-HT, in PD patients (Guldberg et al., 1967; Johansson and Roos, 1967, 1971; Olsson and Roos, 1968; Gottfries et al., 1969; Chase, 1970; Rinne and Sonninen, 1972; Rinne et al., 1973; Davidson et al., 1977; Mayeux et al., 1984, 1986, 1988; Kostić et al., 1987; Tohgi et al., 1993c, 1997; Mashige et al., 1994; Strittmatter et al., 1996; Engelborghs et al., 2003). Other authors report normal CSF 5-HIAA levels (Papeschi et al., 1970, 1972; Godwin-Austen et al., 1971; Granerus et al., 1974; Davidson et al., 1977; Tabaddor et al., 1978; Cramer et al., 1984; Burns et al., 1985; Chia et al., 1993; González-Quevedo et al., 1993; Volicer et al., 1985; Fukuda et al., 1989). Liu et al. (1999) described lower ventricular CSF 5-HIAA levels in patients with rigid-akinetic PD than in patients with tremoric PD, and a negative correlation between CSF 5-HIAA levels and PD severity.

CSF 5-HIAA levels seem to be unchanged by therapy with levodopa (Godwin-Austen et al., 1971; Davidson et al., 1977), bromocriptine (Gumpert et al., 1973), or piribedil (Gumpert et al., 1973), or were found decreased by levodopa therapy (Casati et al., 1973). Gumpert et al. (1973) described an association between relatively low pre-treatment CSF 5-HIAA levels with a good response to levodopa, while Davidson et al. (1977) reported this association with higher CSF 5-HIAA levels, and others found no such relation (Granerus et al., 1974). Tetrahydrobiopterin increased (Dissing et al., 1989), and L-threo-3,4-dihydroxyphenylserine decreased (Maruyama et al., 1994) CSF 5-HIAA levels.

Some authors have described decreased CSF 5-HIAA (Mayeux et al., 1984, 1986, 1988; Mena et al., 1984; Kostić et al., 1987) and 5-HT levels (Mena et al., 1984) in PD patients with depression, while others have described normal CSF 5-HIAA in depressed PD patients (Granerus et al., 1974; Kuhn et al., 1996a), and others still have reported similar CSF 5-HIAA levels in patients with major depression with PD tothose without PD (Pålhagen et al., 2010). Moser et al. (1996) described increased CSF 5-HIAA in PD patients with hallucinations. Iacono et al. (1997) found higher CSF 5-HT and 5-HIAA and lower 5-HTP levels in PD patients with postural instability and gait disorders than in PD patients without these symptoms.

Studies on the correlation of CSF 5-HT metabolite levels and brain 5-HT levels are lacking. The majority of studies report results on CSF 5-HIAA levels, with the controversial results based on short series of cohorts of patients with PD or other parkinsonian syndromes. Current data do not lend support to the role of CSF 5-HIAA as an unequivocal marker of depression linked to PD.

### Noradrenalin (norepinephrine or NE) metabolites

Neurons containing NE in the brain, mainly in the dorsal nuclei of vagus nerve, are involved in the degenerative process of PD (Benito-León et al., 2008). CSF NE levels have been found normal (Turkka et al., 1987; Chia et al., 1993; Kuhn et al., 1996a; Engelborghs et al., 2003) or decreased (Martignoni et al., 1992; Eldrup et al., 1995) in PD patients. CSF levels of 3-methoxy-4-hydroxy-phenylethyleneglycol (MHPG), the main metabolite of NE, have been reported to be normal (Wilk and Mones, 1971; Davidson et al., 1977; Mann et al., 1983; Mena et al., 1984; Hartikainen et al., 1992; Martignoni et al., 1992; Chia et al., 1993; González-Quevedo et al., 1993; Mashige et al., 1994; Kuhn et al., 1996a; Engelborghs et al., 2003) or decreased (Granerus et al., 1974) in PD patients. CSF MHPG levels do not increase either after treatment with levodopa (Wilk and Mones, 1971; Davidson et al., 1977) or with the NE precursor L-Threo-3,4-dihydroxyphenylserine (L-threo-DOPS) (Yamamoto et al., 1986; Teelken et al., 1989), while L-threo-DOPS increases CSF NE levels (Tohgi et al., 1990, 1993d).

Several authors have described a negative correlation between CSF MHPG levels and cognitive functioning (Mann et al., 1983) and bradyphrenia (Mayeux et al., 1987) in PD patients, and others have described a relationship between CSF NE levels with severity of PD assessed by Hoehn & Yahr staging, akinesia scores, and freezing of the gait (Tohgi et al., 1993a). Pålhagen et al. reported decreased CSF MHPG levels in patients with major depression with PD compared to those without PD (Pålhagen et al., 2010).

CSF activity of dopamine-β-hydroxylase (DBH), an enzyme involved in NE synthesis, has been found decreased in PD patients when compared with controls (Matsui et al., 1981; Hurst et al., 1985).

The normality of CSF MHPG levels found in nearly all studies with PD or other parkinsonian syndromes indicates that this is not a useful marker of PD. The correlation between CSF MHPG and brain NE is unknown.

### Acetylcholine (Ach) and related substances

CSF levels of Ach (Duvoisin and Dettbarn, 1967; Welch et al., 1976; Yamada et al., 1996) and its precursor choline (Aquilonius et al., 1972; Welch et al., 1976; Nasr et al., 1993) have been reported to be similar in PD patients to controls with the exception of one study in which lower CSF choline levels were described in PD patients (Manyam et al., 1990).

CSF activity of acetylcholine-esterase (AchE), the main enzyme involved in Ach degradation, has been reported to be similar in PD patients and controls (Jolkkonen et al., 1986; Ruberg et al., 1986; Zubenko et al., 1986; Sirviö et al., 1987; Yoshinaga et al., 1989; Manyam et al., 1990; Hartikainen et al., 1992), although there are studies which have described increased (Ruberg et al., 1986), decreased (Konings et al., 1995), or normal activity (Zubenko et al., 1986; Sirviö et al., 1987) in demented patients, and decreased activity only in those patients with the most severe disease (Hartikainen et al., 1992).

CSF activity of butirylcholine-esterase (BchE) have been found to be similar in PD patients and controls (Ruberg et al., 1986; Sirviö et al., 1987), but increased in demented PD patients in a single study (Ruberg et al., 1986). Data on CSF Ach and related substances are scarce and based on short series of patients, and do not permit valid conclusions.

### Gamma-amino butyric acid (GABA) and other neurotransmitter amino acids

CSF GABA levels in PD patients have been found to be decreased, when compared with controls, by many authors (Lakke and Teelken, 1976; Manyam et al., 1980, 1988; Kuroda et al., 1982; Manyam, 1982; Teychenné et al., 1982; Kuroda, 1983; de Jong et al., 1984; Araki et al., 1986; Tohgi et al., 1991c), while others have found this value to be normal (Enna et al., 1977; Abbott et al., 1982; Bonnet et al., 1987; Perschak et al., 1987; Mally et al., 1997; Engelborghs et al., 2003) or even increased (Jiménez-Jiménez et al., 1996). Manyam and Tremblay (1984) found reduced CSF free GABA levels and normality of conjugated levels. Abbot et al. (Perschak et al., 1987) found decreased CSF GABA levels in PD patients treated with levodopa, but not in “*de novo*” PD patients, while other authors found decreased CSF GABA in untreated PD patients (Manyam, 1982; de Jong et al., 1984), with CSF GABA normal (de Jong et al., 1984; Tohgi et al., 1991c) or slightly decreased (Manyam, 1982) in PD patients under levodopa therapy, suggesting that levodopa increases CSF levels. Teychenné et al. (1982) described low CSF GABA especially in PD patients with poor response to therapy or suffering from “on-off” motor fluctuations.

Normality of CSF glutamate levels has been reported by most investigators (Van Sande et al., 1971; Gjessing et al., 1974; Lakke and Teelken, 1976; Lakke et al., 1987; Perschak et al., 1987; Espino et al., 1994; Jiménez-Jiménez et al., 1996; Kuiper et al., 2000), although 3 groups described decreased CSF glutamate levels (Gründig and Gerstenbrand, 1980; Tohgi et al., 1991c; Mally et al., 1997), while CSF glutamine (the main precursor of glutamate) has been found to be normal (Gjessing et al., 1974; Lakke and Teelken, 1976; Manyam et al., 1988; Jiménez-Jiménez et al., 1996) or increased (Mally et al., 1997).

CSF aspartate levels have been reported as normal (Lakke and Teelken, 1976; Manyam, 1982; Araki et al., 1986; Perschak et al., 1987; Mally et al., 1997; Jiménez-Jiménez et al., 1996; Engelborghs et al., 2003), except in the study by Tohgi et al. (1991c) who reported decreased CSF aspartate; CSF asparagine (the main metabolite of aspartate) has been found normal (Lakke and Teelken, 1976; Manyam, 1982; Araki et al., 1986; Perschak et al., 1987; Jiménez-Jiménez et al., 1996; Mally et al., 1997; Engelborghs et al., 2003).

The results on CSF glycine levels have been reported as normal by most investigators (Gjessing et al., 1974; Perschak et al., 1987; Manyam et al., 1988; Jiménez-Jiménez et al., 1996; Mally et al., 1997; Engelborghs et al., 2003), although two groups found them increased (Lakke and Teelken, 1976; Araki et al., 1986; Lakke et al., 1987), and another decreased (Tohgi et al., 1991c). In agreement with Tohgi et al. (1991c), our group reported lower glycine levels in untreated PD patients when compared with PD patients under levodopa therapy or with controls (Jiménez-Jiménez et al., 1996).

Data regarding other (non-neurotransmitter) amino acids are even more controversial. CSF levels of neutral and basic amino acids have been reported to be both increased (Van Sande et al., 1971; Lakke and Teelken, 1976; Lakke et al., 1987), and decreased (Molina et al., 1997a). Two groups reported decreased (Molina et al., 1997a; Engelborghs et al., 2003) and another increased CSF levels of taurine (Lakke and Teelken, 1976; Araki et al., 1986; Lakke et al., 1987). Ornithine, citruline, and arginine (implicated in the urea cycle, and the two latter in the synthesis of nitric oxide) have been found to be increased (Van Sande et al., 1971; Lakke and Teelken, 1976; Lakke et al., 1987), normal (Kuiper et al., 2000), or decreased (Molina et al., 1997a). Another group described increased CSF levels of total homocysteine but normal ones of free homocysteine in PD patients (Isobe et al., 2005), with an additional increase after treatment with levodopa, while total methionine levels decreased after this therapy (Isobe et al., 2010a).

In general, the results on CSF amino acid levels in PD patients are inconclusive, because they might be influenced by selection of study subjects, sample size, lack of adequate matching between cases and controls in many studies, differences in antiparkinsonian therapy, and differences in study techniques, storage and handling of the samples (Jiménez-Jiménez et al., 1996; Molina et al., 1997a).

### Neuropeptides

Neuropeptides modulate neuronal communication by acting on cell surface receptors. Many of them are co-released with classical neurotransmitters. There have been reports of a number of changes in the concentrations of several neuropeptides in PD brain, which are mainly significant decreases in (Jiménez-Jiménez, 1994): (a) met-enkephalin (MET-ENK), substantia P (SP), and cholecystokinine 8 (CCK-8) in the substantia nigra; (b) MET-ENK and leu-enkephalin (LEU-ENK) in the putamen and globus pallidus; (c) MET-ENK in the ventral tegmental area; (d) SP, somatostatin and neurotensin in the neocortex, and (e) somatostatin and neurotensin in the hippocampus. It is likely that many of these changes are related with dopaminergic deficit, and the only clear relationship between a neuropeptide and a clinical feature of PD is that of somatostatin with the presence of cognitive impairment (Jiménez-Jiménez, 1994). Table 2 summarizes the findings of classical studies on CSF neuropeptide levels in PD patients. Most of these studies enrolled limited series of patients.

**Table 2:** **Alterations in CSF neuropeptide levels in PD patients compared with controls**.

**Neuropeptide**	**References**	**PD patients/ Controls**	**Cerebrospinal fluid levels**
Substantia P (SP)	Pezzoli et al., 1984	12/10	Increased 5-fold
	Cramer et al., 1989	15/9	Normal
	Cramer et al., 1991	23/9	Decreased by 30% (controls were essential tremor patients)
Cholecystokinin-8 (CCK-8)	Lotstra et al., 1985	20/68	Decreased by 50%
Met-enkephalin (MET-ENK)	Pezzoli et al., 1984	12/10	Increased 3-fold in PD patients with slight or moderate disability (*n* = 6)
	Yaksh et al., 1990	8/9	Decreased by 37%
	Baronti et al., 1991	16/19	Decreased by 31.7%
Leu-enkephalin (LEU-ENK)	Liu, 1989	22/19	Increased by 122% in untreated PD patients without further modification by levodopa therapy
Dynorphin A(1-8)	Baronti et al., 1991	16/19	Normal
Somatostatin	Jolkkonen et al., 1986	35/19	Decreased by 22% (*p* < 0.01), especially in demented patients
	Strittmatter and Cramer, 1992	38/12	Decreased by 27.5% (*p* < 0.01)
	Strittmatter et al., 1996	35/11	Decreased *p* < 0.05, similar in untreated vs. treatment with levodopa
	Cramer et al., 1989	15/9	Decreased by 39%
	Dupont et al., 1982	39/29	Decreased by 40%
	Christensen et al., 1984	48/32	Decreased by 40%
	Cramer et al., 1985	50/6	Decreased by 34%(controls were patients with essential tremor)
	Masson et al., 1990	35/11	Decreased (*p* < 0.02), especially in untreated patients and in those with more severe disease
	Jost et al., 1990	68/6	Decreased by 28%
	Hartikainen et al., 1992	35/34	Normal
	Volicer et al., 1986	10/9	Normal
	Beal et al., 1986	6/84	Normal
	Poewe et al., 1990	22/11	Normal in PD patients with dementia (*n* = 11) and without dementia (*n* = 11)
	Espino et al., 1995	23/26	Increased by 47%, especially in demented patients
Neuropeptide Y (NPY)	Martignoni et al., 1992	10/20	Decreased by 31%
	Yaksh et al., 1990	8/9	Normal
Beta-endorphin	Nappi et al., 1985	24/15	Decreased (*p* < 0.005) both in 14 untreated and 10 treated PD patients
	Jolkkonen et al., 1987	36/35	Normal
Arginine-vasopressine (AVP)	Sundquist et al., 1983	11/21	Decreased by 68%
	Olsson et al., 1987	12/32 OND	Decreased by 71%
Vasoactive intestinal peptide (VIP)	Sharpless et al., 1984	19/12	Normal
Delta sleep-inducing peptide (DSIP)	Ernst et al., 1987	9/20	Decreased by 28.7% (Ferrero et al., 1988)
Alpha-melanocyte-stimulating hormone-like	Rainero et al., 1988	9/12	Increased by 2-fold
Diazepam-binding inhibitor	Ferrero et al., 1988	25/82	Increased by 42.5% (80% in depressed PD patients and normal in non-depressed PD patients
	Ferrarese et al., 1990	28/10	Decreased by 50% in PDD (*n* = 14), normal in PDND (*n* = 14)
Neurokinin A	Galard et al., 1992	12/11	Decreased by 24%
Corticotropin-releasing hormone (CRH)	Suemaru et al., 1995	10/5	Normal
ACTH	Nappi et al., 1985	24/15	Normal
Beta-lipotropine	Nappi et al., 1985	24/15	Normal
Angiotensin converting enzyme (ECA)	Konings et al., 1994	88 PDND/18 PDD/20	Increased in PDND patients under levodopa therapy (*p* < 0.05). Normal in untreated PDND and in PDD
	Zubenko et al., 1985	10 PDD/30	Decreased by 27% in demented PD patients
	Zubenko et al., 1986	15/10	Decreased by 24%
Chromogranin A and B and secretogranin II	Eder et al., 1998	8/29	Normal

OND, other neurological diseases; PDD, Parkinson's disease demented; PDND, Parkinson's disease non-demented.

In recent years, there has been increased interest in the possible role of orexin-A/hypocretin-1, a neuropeptide hormone implicated in the pathogenesis of narcolepsia, on the development of excessive daytime sleepiness in PD patients. Since the first report by Drouot et al. (2003), who described decreased ventricular CSF orexin levels in PD patients, which were related with the severity of the disease, other authors have confirmed decreased CSF orexin in PD (Fronczek et al., 2007; Asai et al., 2009) and in other neurodegenerative parkinsonisms (Yasui et al., 2006), and the relation of CSF orexin with severity of PD (Asai et al., 2009), and with the presence of sleep attacks (Asai et al., 2009). In contrast, Compta et al. (2009a) found no significant differences in CSF orexin levels between demented PD patients, non-demented PD patients, and healthy controls, and found no relation between CSF orexine and Epworth sleepiness scale or Mini-Mental State Examination. Drouot et al. (2011) found a lack of association between low ventricular CSF orexin and sleepiness in PD, and a relation between high levels of orexin-A in PD associated with loss of REM muscle atonia (Bridoux et al., 2013), while Wienecke et al. (2012) reported association between low CSF orexin levels and sleepiness in PD. Finally, Pålhagen et al. (2010) described similar CSF orexin levels in patients with major depression with or without concomitant PD. The results regarding orexin A are controversial, and await confirmation.

### Other neurotransmitters

Pisani et al. (2005, 2010) found increased CSF levels of the endogenous cannabinoid anandamide in untreated PD patients, which were unrelated to the severity of the disease (Pisani et al., 2005) and reversed by chronic dopaminergic replacement (Pisani et al., 2010). Zhou et al. (1997) found decreased CSF β-phenylethylamine (PEA) levels in PD patients which were correlated negatively with Hoehn & Yahr stage.

### Cyclic nucleotides

These compounds act as intracellular second messengers of neurotransmitters or other compounds such as nitric oxide (NO). The most important are cyclic adenosine 3′5′ monophosphate (cAMP) and cyclic guanosine 3′5′ monophosphate (cGMP). Belmaker et al. (1978) reported a 40–50% decrease of CSF cAMP and an 80–90% decrease of CSF cGMP levels in PD patients who were not related with levodopa therapy. Decreased CSF cAMP levels in PD have also been reported in another study (Volicer et al., 1986), while others found this value to be normal (Cramer et al., 1973, 1984; Covicković-Sternić et al., 1987; Oeckl et al., 2012), both in PD patients with and without dementia (Oeckl et al., 2012). Four further studies described normal CSF cGMP levels (Volicer et al., 1986; Covicković-Sternić et al., 1987; Ikeda et al., 1995; Oeckl et al., 2012), while another found a non-significant trend toward higher CSF cGMP levels in PD patients when compared with controls and higher levels in levodopa-treated PD patients compared with those without levodopa treatment (Navarro et al., 1998).

### Biopterin derivatives and other cofactors

Biopterins act as cofactors for aromatic amino acid hydroxylases, which produce a number of neurotransmitters including DA, NE, epinepherine, and 5-HT and are also required for the production of NO. CSF levels of neopterin and biopterin have been found decreased in PD patients by several groups, especially in those with early-onset PD (Fujishiro et al., 1990; Furukawa et al., 1992), and in carriers of the *PARK8* mutation (Koshiba et al., 2011), which was negatively correlated with duration of illness in those patients with akinetic-rigid PD (Furukawa et al., 1991). In contrast, another group found increased CSF neopterin in PD (Widner et al., 2002).

CSF concentration of hydroxylase cofactor, predominantly composed of tetrahydrobiopterin (BH_4_), has also been found decreased (Williams et al., 1980a,b).

Thiamine is an essential cofactor for several important enzymes involved in brain oxidative metabolism. Our group found normal CSF levels of thiamine-diphosphate, thiamine-monophosphate, free thiamine, and total thiamine in PD patients (Jiménez-Jiménez et al., 1999).

## Endogenous neurotoxins

One of the classical etiological hypotheses of PD is related with the presence of endogenous substances which share structural similarities with 1-methyl-4-phenyl-1,2,3,6-tetrahydropyridine (MPTP), a neurotoxin that induces a parkinsonism resembling PD.

Moser et al. (Moser and Kömpf, 1992; Moser et al., 1995) identified two tetrahydroisoquinolin (TIQ) derivatives in the CSF of PD patients, but not in healthy controls, 2-methyl and 1-methyl-6,7-dihydroxy1,2,3,4-TIQ (2-MDTIQ and 1-MDTIQ or salsolinol). This group described a relation between high salsolinol levels and the presence of visual hallucinations (Moser et al., 1996), and reported an increased HVA/3OMD ratio in PD patients in which 2-MDTIQ was detected when compared with those PD in which it was not detectable.

CSF salsolinol levels have been reported to be increased in PD patients compared with controls by other groups (Maruyama et al., 1996; Antkiewicz-Michaluk et al., 1997; Krygowska-Wajs et al., 1997; Naoi and Maruyama, 1999), especially in demented PD patients (Antkiewicz-Michaluk et al., 1997), and in those patients with more severe parkinsonism (Krygowska-Wajs et al., 1997), although other authors have described a trend toward decrease in CSF salsolinol levels with the progression of the disease (Maruyama et al., 1999). In contrast, another group reported similar CSF salsolinol (Müller et al., 1999a,b), but higher levels of harman and norharman β-carbolines (structural analogs of MPTP as well) in PD patients than in controls (Kuhn et al., 1996b). CSF levels of 1-benzyl-1,2,3,4-TIQ have also been found by another group to be increased (Kotake et al., 1995).

Matsubara et al. (1995) measured β-carbolinium cations (BC+s) in the lumbar CSF of 22 PD patients and 11 age-matched controls, and found the 2,9-dimethylnorharmanium cation (2,9-Me2NH+) in 12 PD patients but not in controls. This group described decreased activity of nicotinamide N-methyltranserase (NNMT), an enzyme that catalyzes the N-methylation of nicotinamide and other pyridines in the CSF of younger PD patients compared with younger controls, and a trend toward decrease with aging in PD patients (Aoyama et al., 2001).

The results of studies on neurotoxins related with the risk for PD are based on small series and are not conclusive.

## Oxidative stress markers

**Figure 1:**
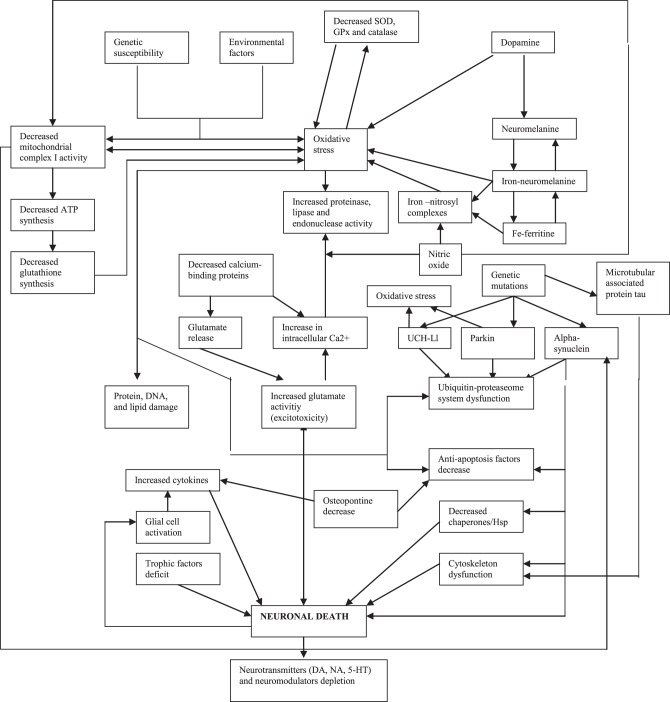
**Pathogenical mechanisms proposed for Parkinson's disease (modified from Alonso-Navarro et al., 2008)**.

Because there is much evidence on the contribution of oxidative stress in the pathogenesis of PD (Figure 1) (Alonso-Navarro et al., 2008), the measurement of oxidative stress markers and substances related with oxidative and defense against oxidative phenomena in the CSF of PD patients is useful. Data regarding lipid peroxidation markers are controversial, while DNA oxidation markers have been found to be increased (Table 3).

**Table 3:** **Alterations in the CSF levels of oxidative stress markers and substances related with oxidative stress in PD patients compared with controls**.

		**References**	**PD/Controls**	**Cerebrospinal fluid levels**
Lipid peroxidation markers	Malonyl-dialdehyde (MDA)	Ilić et al., 1998	31/16	Increased (*p* < 0.001)
		Ilic et al., 1999	33/16	Increased (*p* < 0.001)
		Shukla et al., 2006	21/20	Normal
	(E)-4-hydroxynonenal (HNE)	Selley, 1998	10/10	Increased 4-fold
	Low density lipoprotein (LDL) oxidation products	Buhmann et al., 2004	70/60 OND/31 HC	Increased 3-fold with –SH decreased 1.5-fold
	Schiff bases, conjugated dienes, oxidized proteins, and aldehyde polymers	Boll et al., 2008	22/41	Increased 1,5 fold (Isobe et al., 2010b)
DNA oxidation markers	8'-hydroxy-2'deoxyguanine (8-OHdG)	Kikuchi et al., 2002	48/22	Increased (*p* < 0.0001)
		Isobe et al., 2010b	20/20	Increased (*p* < 0.0001)
	8-hydrosyguanosine (8-OHG)	Kikuchi et al., 2002	48/22	Increased
		Abe et al., 2003	24/15	Increased 3-fold (*p* < 0.001)
	8-OHdG/8-OHG ratio	Kikuchi et al., 2002	48/22	Increased 2-fold (*p* < 0.0005)
Transition metals and related proteins	Iron	Campanella et al., 1973	13/5	Normal
		Pall et al., 1987	24/34	Normal
		Gazzaniga et al., 1992	11/22	Normal
		Takahashi et al., 1994	20/25	Normal
		Pan et al., 1997	NS/NS	Normal
		Jiménez-Jiménez et al., 1998	37/37	Normal
		Hozumi et al., 2011	20/15	Normal
		Forte et al., 2004	26/13	Decreased (*p* < 0.05)
		Alimonti et al., 2007	42/20	Decreased (*p* < 0.05)
		Qureshi et al., 2006	36/21	Increased
	Ferritin	Campanella et al., 1973	13/5	Normal
		Dexter et al., 1990	26/11	Normal
		Pall et al., 1990	24/21	Normal
		Kuiper et al., 1994a	72 PDND/15 PDD/20 HC	Normal
	Transferrin	Loeffler et al., 1994	12/11	Normal
	Copper	Campanella et al., 1973	13/5	Normal
		Gazzaniga et al., 1992	11/22	Normal
		Takahashi et al., 1994	20/25	Normal
		Pan et al., 1997	NS/NS	Increased (*p* < 0.05)
		Jiménez-Jiménez et al., 1998	37/37	Normal
		Forte et al., 2004	26/13	Normal
		Alimonti et al., 2007	42/20	Normal
		Qureshi et al., 2006	36/21	Normal
		Boll et al., 2008	22/41	Increased 2-fold
		Pall et al., 1987	24/34	Increased (*p* < 0.001)
		Hozumi et al., 2011	20/15	Increased 2-fold (*p* < 0.01)
		Boll et al., 1999	49/26 (35 PD untreated)	Increased 1,5 fold
	Ceruloplasmin	Campanella et al., 1973	13/5	Normal
		Loeffler et al., 1994	12/11	Normal
	Ferroxidase	Boll et al., 2008	22/41	Decreased activity by 20%
		Boll et al., 1999	49/26 (35 PD untreated)	Decreased activity by 1.5-fold
	Manganese	Gazzaniga et al., 1992	11/22	Normal
		Pan et al., 1997	NS/NS	Normal
		Jiménez-Jiménez et al., 1998	37/37 26/13	Normal Normal
		Forte et al., 2004		
		Alimonti et al., 2007	42/20	Normal
		Hozumi et al., 2011	20/15	Increased 1.5-fold (*p* < 0.05)
	Zinc	Takahashi et al., 1994	20/25	Normal
		Pan et al., 1997	NS/NS	Normal
		Forte et al., 2004	26/13	Normal
		Jiménez-Jiménez et al., 1998	37/37	Decreased (*p* < 0.05)
		Qureshi et al., 2006	36/21	Decreased
		Hozumi et al., 2011	20/15	Increased 3-fold (*p* < 0.01)
Other metals	Selenium	Takahashi et al., 1994	20/25	Normal
		Qureshi et al., 2006	36/21	Increased
		Aguilar et al., 1998	28/43	Increased only in untreated PD patients (*p* < 0.01)
	Chromium	Aguilar et al., 1998	28/43	Normal
		Alimonti et al., 2007	42/20	Decreased by 50%
	Magnesium	Hozumi et al., 2011	20/15	Normal
		Forte et al., 2004	26/13	Normal
		Alimonti et al., 2007	42/20	Normal
	Calcium	Pan et al., 1997	NS/NS	Normal
		Forte et al., 2004	26/13	Normal
		Alimonti et al., 2007	42/20	Normal
	Aluminum	Forte et al., 2004	26/13	Decreased (*p* < 0.05)
		Alimonti et al., 2007	42/20	Normal
	Silicon	Forte et al., 2004	26/13	Normal
		Alimonti et al., 2007	42/20	Decreased (*p* < 0.05)
	Cobalt	Alimonti et al., 2007	42/20	Decreased (*p* < 0.05)
	Tin	Alimonti et al., 2007	42/20	Decreased (*p* < 0.05)
	Lead	Alimonti et al., 2007	42/20	Decreased by 50%
	Various	Alimonti et al., 2007	42/20	Normal levels of barium, bismuth, cadmium, mercury, molibdenum, nickel, antimony, strontium, thallium, vanadium, wolfram, and zirconium
Nitric oxide metabolites/nitroxidative stress	Nitrates	Ikeda et al., 1995	11/17	Normal
		Molina et al., 1996	31/38	Normal
		Kuiper et al., 1994b	103/20	Decreased
		Boll et al., 2008	22/41	Increased 2-fold
	Nitrites	Ikeda et al., 1995	11/17	Normal
		Ilic et al., 1999	33/?	Normal
		Kuiper et al., 1994b	103/20	Normal
		Boll et al., 2008	22/41	Increased 2-fold
		Qureshi et al., 1995	16/14	Increased 2-fold both in untreated (*n* = 6) and in levodopa-treated (*n* = 10) PD patients. Controls were young
	Nitrotyrosine-containing proteins	Fernández et al., 2013	54/40	Increased (*p* < 0.01)
		Aoyama et al., 2000	10/6	Increased 1.8-fold
Antioxidant enzymes or substances	Total superoxide-dismutase (SOD)	Marttila et al., 1988	26/26 OND	Normal
		De Deyn et al., 1998	12/58	Normal
	Cu/Zn-SOD (SOD-1)	Ilić et al., 1998	31/16	Increased (*p* < 0.05)
		Ilic et al., 1999	33/16	Increased (*p* < 0.05)
		Boll et al., 2008	22/41	Decreased (*p* = 0.021)
	Mn-SOD (SOD-2)	Aoyama et al., 2000	10/6	Normal
	Catalase	Marttila et al., 1988	26/26 OND	Normal
	Glutathione peroxidase (GPx)	Marttila et al., 1988	26/26 OND	Normal
	Glutathione reductase (GR)	Ilić et al., 1998	31/?	Increased
		Ilic et al., 1999	33/?	Increased
	Reduced glutathione (GSH)	Marttila et al., 1988	26/26 OND	Normal
		Tohgi et al., 1995b	22/15	Increased (*p* < 0.02) in L-dopa treated patients (*n* = 8)
		Konings et al., 1999	71 PD/13 PDND/21 HC	Normal
	Oxidized glutathione (GSSG)	LeWitt et al., 2013	48/57	Decreased (*p* < 0.01)
		Tohgi et al., 1995b	22/15	Decreased (*p* < 0.001) in untreated patients (*n* = 14)
	Alpha-tocopherol (vitamin E)	Buhmann et al., 2004	70/60 OND/31 HC	Decreased by 44–48%
		Tohgi et al., 1995b	22/15	Normal
		Molina et al., 1997b	34/47	Normal
	Alpha-tocopherol-quinone	Tohgi et al., 1995b	22/15	Decreased (*p* < 0.001) in untreated patients (*n* = 15)
	Urate	Tohgi et al., 1993e	11/14	Normal
		Constantinescu et al., 2013	6/18	Normal
		Ascherio et al., 2009	713/0	Relation of higher CSF levels of urate with slower rates of clinical decline
	Xantine (uric acid precursor)	LeWitt et al., 2011	217/26	Normal
	Ascorbate	Buhmann et al., 2004	70/60 OND/31 HC	Normal
	Carnitine	Jiménez-Jiménez et al., 1997	29/29	Normal
	Oxidized coenzyme Q10/total Q10 ratio	Isobe et al., 2010b	20/20	Increased 18% (*p* < 0.05)
		Isobe et al., 2007	20/20	Increased 18% (*p* < 0.05)
	Osteopontine	Maetzler et al., 2007	30/30	Increased 2-fold (*p* < 0.002)

OND, other neurological controls; HC, healthy controls; PDND, Parkinson's disease non-demented.

Transition metals such as iron, copper, and manganese, act as prooxidant agents, although copper is also essential for the antioxidant function of the protein ceruloplasmin, and copper and manganese are constituents of the cytosolic Cu^+2^/Zn^+2^ and the mitochondrial Mn^+2^-superoxide-dismutases (SOD, protective against oxidative processes). Zinc has antioxidant activity and is a constituent of Cu^+2^/Zn^+2^-SOD (Jiménez-Jiménez et al., 1998). The results of studies with CSF levels of iron and copper are controversial (Table 3), but a recent meta-analysis showed similar values in PD patients to controls (Mariani et al., 2013), thus suggesting that these metals are not useful as markers of PD.

Together with its role in glutamate excitotoxity, NO could contribute to oxidative stress mechanisms in the pathogenesis of PD by interacting with ferritin to release iron, inducing mitochondrial complex I damage (Molina et al., 1998), and by inducing nitrosylation of proteins (Fernández et al., 2013). However, studies on CSF levels of nitrates and nitrites have given controversial results (Table 3).

Among other antioxidant enzymes and substances (Table 3), one study involving an important number of early PD patients showed the relationship between the presence of relatively higher levels of urate and the slower rates of clinical decline (Ascherio et al., 2009), despite the fact that CSF urate levels were found to be similar in PD patients and controls in the same study.

## Inflammatory and immunological markers

CSF interleukin (IL) 1-β levels were found to be normal in one study (Pirttila et al., 1994) and increased in three (Blum-Degen et al., 1995; Mogi et al., 1996a; Mogi and Nagatsu, 1999), CSF IL-2 normal (Blum-Degen et al., 1995) or increased (Mogi et al., 1996a; Mogi and Nagatsu, 1999), IL-4 increased (Mogi and Nagatsu, 1999), and CSF IL-10, IL-12, and interferon-gamma levels have been reported to be similar in PD patients and controls (Rota et al., 2006). CSF IL-6 levels have been found to be decreased in PD patients with major depression in comparison with patients with major depression without PD in one study (Pålhagen et al., 2010), while another 4 found higher CSF IL-6 in PD patients than in healthy controls (Blum-Degen et al., 1995; Mogi et al., 1996a; Müller et al., 1998; Mogi and Nagatsu, 1999), and in one of them CSF IL-6 was correlated with PD severity (Müller et al., 1998). CSF tumor necrosis α (TNF-α) levels have been found to be increased (Mogi et al., 1994; Mogi and Nagatsu, 1999), leukotriene 4 (Irkeç et al., 1989), and α-1-antichymotrypsin normal (Pirttila et al., 1994), and β-2-microglobuline decreased in PD (Mogi et al., 1989; Mogi and Nagatsu, 1999). The CSF levels of the cytokine fractalkine have been found to be normal in PD patients and increased in multiple system atrophy (MSA), and Flt3 ligand normal in these two diseases (Shi et al., 2011). The presence of certain syalilated isoforms of Serpin A1 in the CSF has been related with the development of dementia in PD patients (Jesse et al., 2012).

CSF levels of pros-methylimidazol acetic acid, an isomer of the histamine metabolite tele-methylimidazol acetic acid, have been found to be decreased in PD (Prell et al., 1991), and were highly positively correlated with the severity of the disease (Prell and Green, 1991).

CSF complement 3 (C_3_) and factor H (FH) levels were reported to be normal in one study (Wang et al., 2011), while another described a decrease in several isoforms of C_3b,_C_4b_, FH, and factor B (Finehout et al., 2005), and another normal C_4d_ (Yamada et al., 1994). CSF levels of heat shock proteins Hsp65 and Hsp70 have been found to be increased (Fiszer et al., 1996), and PD patients have shown higher HLA-DR expression in CSF monocytes in comparison with controls (Fiszer et al., 1994a).

Oligoclonal IgG bands have not been detected in the CSF of PD patients (Chu et al., 1983), but antibodies against DA neurons have been detected in 78% of PD patients and in only 3% of controls (Carvey et al., 1991), and the CSF of PD patients has shown a higher proportion of gamma-delta-T+ cells than in controls (Fiszer et al., 1994b).

The results of studies on inflammatory and immunological markers in PD have a low number of patients and controls enrolled, and are inconclusive.

## Growth and neurotrophic factors

CSF Brain Derived Neurotrophic Factor (BDNF) levels have been found to be similar in PD patients with major depression to those in patients with major depression without PD in one study (Pålhagen et al., 2010), while another described this value as increased in PD patients compared with controls (Salehi and Mashayekhi, 2009). CSF Transforming Growth Factor α (TGF-α) has been found to be increased in juvenile parkinsonism (Mogi and Nagatsu, 1999). TGF-β1 has been found to be increased (Mogi et al., 1995, 1996a; Vawter et al., 1996; Mogi and Nagatsu, 1999) or normal (Rota et al., 2006), and TGF-β2 increased (Vawter et al., 1996). CSF insulin-like growth factor-1 (IGF-1) and IGF binding proteins (IGFBPs) expression is increased in PD patients (Mashayekhi et al., 2010). Finally, a single study found a non-significant trend toward increased CSF levels of neuroregulins (which belong to the Epidermal Growth Factor or EGF family) in PD patients (Pankonin et al., 2009). The results of studies on growth and neurotrophic factors in PD, involving a low number of patients and controls, do not permit definitive conclusions.

## Proteins involved in the pathogenesis of parkinson's disease

### Microtubular associated protein *Tau* (MAPT)

Because *MAPT* gene is one of the main genes involved in the risk for PD (Alonso-Navarro et al., 2014), the measurement of CSF protein *tau* levels are hypothetically useful as a marker of this disease. *Tau* protein is important for maintaining the stability of axonal microtubules involved in the mediation of fast axonal transport of synaptic constituents. Hyperphosphorylation of *tau* causes reduces binding affinity for microtubules, leading to their malfunction. Following neuronal damage, tau is released into extracellular space and may be increased in the CSF. *Tau* is an important component of the neurofibrillary tangles (pairwise, helical protein filaments which are found in the cytoskeleton or neuronal cells in Alzheimer's disease (AD) brains. CSF *tau* protein levels are increased in AD patients, and so are a useful marker for this disease. The high risk of PD patients of developing cognitive impairment or dementia patients makes measurement of CSF *tau* reasonable as a possible marker of this disease.

Many studies have shown similar CSF total *tau* and phosphorylated *tau* (phospho*tau*) in PD patients to controls (Blennow et al., 1995; Molina et al., 1997c; Jansen Steur et al., 1998; Sjögren et al., 2002; Mollenhauer et al., 2006; Parnetti et al., 2008, 2011, 2014a,b; Ohrfelt et al., 2009; Compta et al., 2009b; Alves et al., 2010; Montine et al., 2010; Aerts et al., 2011; van Dijk et al., 2013a; Herbert et al., 2014). Several of these studies have shown increased CSF *tau* in demented PD patients (Mollenhauer et al., 2006; Compta et al., 2009b). The 33 KDa/55 KDa *tau* isoforms ratio have also been found to be normal in PD (Borroni et al., 2008, 2009), but decreased in progressive supranuclear palsy (PSP), and normal in patients with diffuse Lewy body disease (DLBD), demented PD patients (PDD), AD, and frontotemporal dementia (FTD) (Borroni et al., 2008, 2009).

Some authors have found decreased CSF total *tau* and phospho*tau* levels when compared with controls (Mollenhauer et al., 2011; Shi et al., 2011; Kang et al., 2013) and similar levels in PD to PSP, DLBD, and MSA (Mollenhauer et al., 2011), while others found higher CSF *tau* in DLBD compared with PDD patients (Andersson et al., 2011), and still others higher CSF total *tau* in MSA than in PD patients (Herbert et al., 2014). Hall et al. (2012) reported decreased CSF total *tau* and normal phospho*tau* both in PD and PDD, while total *tau* was increased in CBD and normal in PSP, DLBD, and MSA, and phospho*tau* was decreased in PSP and MSA in comparison with controls.

Přikrylová Vranová et al. (2010) found increased CSF *tau* levels in PD patients with less than 2 years of evolution, and increased CSF *tau* levels which were higher in patients with PDD than in PD, and in PD than in controls, and similar CSF *tau* in DLDB than in controls (Vranová et al., 2014). This group and others found increased CSF total *tau* levels in patients with non-tremor variants of PD as compared to tremor-dominant PD and controls (Jellinger, 2012; Přikrylová Vranová et al., 2012). Compta et al. (2011) described increased CSF *tau* levels in PD patients carrying the allele rs242557A. Siderowf et al. (2010) showed a lack of association between baseline CSF *tau* levels and cognitive decline in PD patients. Patients with corticobasal degeneration (CBD) and PSP have shown higher CSF total and phospo*tau* levels (Aerts et al., 2011), and patients with DLBD showed similar CSF *tau* levels to PD patients in one study (Ohrfelt et al., 2009), while other authors found higher CSF *tau* levels in AD than in DLBD, in DLDB higher than in PDD, and in PDD higher than in PD (Parnetti et al., 2008).

Baseline CSF levels of total and phospho*tau* in the DATATOP study, involving 403 early PD patients, were negatively correlated with disease progression assessed with the Unified PD Rating Scale (UPDRS) (Zhang et al., 2013).

Beyer et al. (2013) reported a lack of correlation between CSF levels of total and phospho*tau*, and ventricular size in 73 non-demented PD patients and 18 PD patients with mild cognitive impairment.

The results of the studies reported on CSF *tau* levels in PD are summarized in Table 4. Although these results are not conclusive, CSF *tau* levels could be related to the progression of the disease (Zhang et al., 2013), and to the preservation of cognitive function in PD patients (Stewart et al., 2014).

**Table 4:** **Results of studies on CSF tau and phosphotau levels in PD, other parkinsonian syndromes and controls**.

**References**	**Cases/Controls**	**Main findings**
Blennow et al., 1995	44 AD, 31 controls, 17 VAD, 11 FTD, 15 PDND, major depression	CSF total tau and phosphorylated tau (phosphotau) higher in AD than in controls, VAD, FTD, PDND, and major depression (PDND similar than controls)
Molina et al., 1997c	26 PDND, 25 controls	CSF total tau similar in PD and controls
Jansen Steur et al., 1998	115 PD (48 with MMSE lower than 25) 15 controls	CSF total and phosphotau similar in PD (not related with MMSE scores) and controls
Sjögren et al., 2002	19 AD, 14 FTD, 11 ALS, 15 PD, 17 controls	CSF total tau and phosphotau increased in AD compared with FTD (*p* < 0.001), ALS (*p* < 0.001), PD (*p* < 0.001), and controls (p < 0.001)
Mollenhauer et al., 2006	73 PDD, 23 PDND, 41 controls (non-demented neurological patients)	CSF total tau significantly higher in PDD than in PDND and controls. This observation was most marked (*p* < 0.05) in a subgroup of patients with PDD carrying the apolipoprotein genotype epsilon3/epsilon3
Parnetti et al., 2008	19 DLBD, 18 PDD, 23 AD, 20 PDND, 20 controls	CSF total tau of DLBD patients significantly lower than in AD patients, but twofold to threefold higher than in PDD, PDND, or control subjects
		CSF total tau levels similar in PDD and PDND
		Phosphotau increased in the AD group only
Borroni et al., 2008	21 PSP, 20 CBD, 44 FTD, 29 AD, 10 PDND, 15 DLBD, 27 controls	CSF tau 33/55 kDa ratio significantly reduced in PSP when compared to controls and to patients with other neurodegenerative conditions
		CSF tau 33/55 kDa ratio decrease correlated significantly with brainstem atrophy
Borroni et al., 2009	78 patients with neurodegenerative disorders and 26 controls	CSF tau 33/55 kDa ratio significantly decreased in patients with PSP (0.46 ± 0.16) when compared to healthy controls (*p* = 0.002), AD (*P* < 0.001), FTD, CBD, PD, and DLBD (values in PD similar to those of controls)
Ohrfelt et al., 2009	66 AD, 15 PD, 15 DLBD, 55 controls	CSF total tau and phosphotau increased significantly in AD, similar levels in PD, DLBD, and controls
Compta et al., 2009b	20 PDND, 20 PDD, 30 controls patients	CSF total tau and phosphotau higher in PDD than in PDND and controls (*P* < 0.05). High CSF total tau and phospho-tau were associated with impaired memory and naming
Alves et al., 2010	109 PDND, 36 controls, 20 mild AD	CSF total tau and phosphotau similar in PD and controls
		CSF tau did not correlate with cognitive measures
Montine et al., 2010	150 controls (115 >50 years; 24 amnestic Mild Cognitive Impairment (aMCI), 49 AD, 49 PD, 11 PDD 62 PD-CIND (cognitive imparment non-demented)	CSF total tau and phospho181-tau significantly increased in AD and aMCI in comparison with the other groups
		Total tau similar in PDD, PDD and PD-CIND and controls
		Phospho181-tau slightly decreased when compared with controls >50 years
Přikrylová Vranová et al., 2010	32 PD, 30 controls	CSF total tau and total tau/beta-amyloid (1-42) ratio higher in PD than in controls (*p* = 0.045 and 0.033, respectively)
Siderowf et al., 2010	45 PD, longitudinal follow-up at least 1 year	No association between CSF total tau and phospo181-tau and cognitive decline
Aerts et al., 2011	21 PSP, 12 CBD, 28 PD, 49 controls	CSF total tau CBD > PSP > PD = controls
		CSF phospotau CBD > PSP = PD = controls
Parnetti et al., 2011	38 PD, 32 DLBD, 48 AD, 31 FTD, 32 controls with other neurological diseases (*n* = 32)	CSF total tau and phosphotau AD > FTD > DLBD = PD = controls
Shi et al., 2011	137 controls, 126 PD, 50 AD and 32 MSA	CSF total tau and phosphotau AD > controls > PD = MSA
Mollenhauer et al., 2011	Cross-sectional cohort: 51 PD, 29 MSA, 55 DLBD, 62 AD, and 72 neurological controls	CSF total tau AD > DLBD > PD = controls = MSA
Mollenhauer et al., 2011	Validation cohort: 275 PD, 15 MSA, 55 66 DLBD, 8 PSP,22 normal pressure hydrocephalus (NPH) and 23 neurological controls	CSF total tau MSA < DLBD = PD < DLBD < controls
Andersson et al., 2011	47 DLBD, 17 PDD (*n* = 17)	CSF total-tau higher in DLBD than in PDD
		CSF phosphotau similar in DLBD and PDD
Compta et al., 2011	38 PD patients (19 PDD, 19 PDND). All cases were genotyped for a series of tau gene polymorphisms rs1880753, rs1880756, rs1800547, rs1467967, rs242557, rs2471738, and rs7521	The A-allele rs242557 polymorphism was the only tau gene variant significantly associated with higher CSF tau and phospho-tau levels, under both dominant and dose-response model. This association depended on the presence of dementia, and was only observed in individuals with low (<500 pg/mL) CSF Aβ levels
Hall et al., 2012	90 PDND, 33 PDD, 70 DLBD, 48 AD, 45 PSP, 48 MSA, 12 CBD, 107 controls	CSF total tau AD > MSA = CBD > PSP = Controls = DLBD > PDND = PDD
		CSF phosphotau increased in AD, AD > PDD = DLBD = controls = CBD > PDND > PSP = MSA
Přikrylová Vranová et al., 2012	48 PD (17 early-onset PD, 15 tremor dominant, 16 non-tremor-dominant), 19 neurological controls, 18 AD	CSF tau and index tau/amiloid beta42 increased in non-tremor-dominant PD compared with controls, and other PD groups, and siminar to those of AD
Jellinger, 2012	12 PD (6 tremor-dominant PD and 6 non-tremor-dominant PD), 27 AD, 17 controls	CSF total tau higher in AD compared with the other groups, and higher in tremor-dominant PD compared with non-tremor dominant PD and controls
van Dijk et al., 2013a	52 PD, 50 controls	CSF total tau and phosphotau similar in PD and controls
Kang et al., 2013	63 PD, 39 controls	CSF total tau and phosphotau181 significantly lower in PD than in controls
Zhang et al., 2013	403 early stage PD patients enrolled in the DATATOP study	Baseline CSF phosphotau/total tau and phosphotau/amyloid beta significantly and negatively correlated with the rates of the Unified Parkinson Disease Rating Scale change
Beyer et al., 2013	73 PDND, 18 PD with mild cognitive impairment	No associations between CSF total tau and phosphotau and hippocampal atrophy
Herbert et al., 2014	43 PD, 23 MSA, 30 controls	CSF total tau significantly lower in PD than in MSA, but similar to those of controls
		CSF phosphotau similar in PD, MSA and controls
Parnetti et al., 2014a	71 PD (8 of 44 carriers of a mutation in the beta-glucocerebrosidase gene (*GBA1*) 45 controls with other neurological disases	CSF total tau and phosphotau similar in PD and controls
Parnetti et al., 2014b	44 PD and 25 controls with other neurological diseases	CSF total tau and phosphotau similar in PD and controls, and unrelated with prognosis and cognitive impairment
Vranová et al., 2014	27 PDND, 14 PDD, 14 DLBD, 17 AD 24 controls	CSF total tau AD > PDD > PDND > DLBD = controls

AD, Alzheimer's disease; PD, Parkinson's disease; VAD, vascular dementia; FTD, frontotemporal dementia; PDND, PD non-demented; PD, PD demented; MMSE, MiniMental State Examination; DLBD, diffuse Lewy body disease; PSP, progressive supranuclear palsy; CBD, corticobasal degeneration; MSA, multiple system atrophy; aMCI, Amnestic Mild Cognitive Impairment; PD-CIND, PD with cognitive imparment non-demented; NPH, normal pressure hydrocephalus.

### Alpha-synuclein

Alpha-synuclein (α-synuclein) is a 140 amino acid-long presynaptic protein, which is the major component of the Lewy bodies (the neuropatologic hallmark of PD), and has been implicated in the pathogenesis of PD and in synucleinopathies such as MSA and DLBD. Mutations of the α-*synuclein* (*SNCA*) gene are related with early-onset monogenic familial PD and are associated with increased risk for sporadic PD (Alonso-Navarro et al., 2014). Although early studies failed to detect the native form of α-synuclein in the CSF of PD and control patients (Jakowec et al., 1998), later studies have detected monomeric SNC in the CSF, with similar levels in PD patients and controls (Borghi et al., 2000). Several studies have found similar CSF total α-synuclein levels in PD patients and in controls (Woulfe et al., 2002; Ohrfelt et al., 2009; Park et al., 2011; Parnetti et al., 2011; Tateno et al., 2012) and others decreased CSF α-synuclein in PD (Tokuda et al., 2006; Hong et al., 2010; Mollenhauer et al., 2011, 2013; Hall et al., 2012; Wang et al., 2012; Kang et al., 2013; Wennström et al., 2013; Parnetti et al., 2014a,b; Mondello et al., 2014; van Dijk et al., 2014), DLBD (Parnetti et al., 2011; Wennström et al., 2013), MSA (Wang et al., 2012; Mondello et al., 2014), and PSP (Wang et al., 2012). Four studies have reported increased CSF oligomeric α-synuclein levels in PD compared with controls (Tokuda et al., 2010; Park et al., 2011; Parnetti et al., 2014a,b), and one of them showed increased CSF α-Syn in PD patients compared with patients with PSP and AD (Tokuda et al., 2010). Wang et al. (2012) found increased CSF levels of the phosphorylated α-synuclein phospho-Ser129 (PS-129) in PD patients when compared with controls, but lower levels in MSA and PSP of this protein than in PD patients and controls.

Aerts et al. (2012) found similar CSF α-synuclein levels in PD patients to DLBD, PSP, and MSA. Hall et al. (2012) found higher CSF α-synuclein in PSP than in PD, PDD, DLBD, and MSA. Tateno et al. (2012) reported similar CSF α-synuclein levels in PD, MSA, DLBD, and controls but higher CSF α-synuclein levels in AD patients, while Ohrfelt et al. (2009) found higher CSF α-Syn levels in AD than in DLDB and PD, and in DLBD than in PD patients. Foulds et al. (2012) found similar post-mortem CSF total α-synuclein levels in PD, MSA, DLBD, and PSP, but increased CSF levels of phosforylated oligomers in MSA.

van Dijk et al. (2014) reported a lack of relation between CSF α-synuclein levels and striatal dopaminergic deficit measured by dopamine transporter binding and single photon emission computed tomography. In addition, a recent study by Shi et al. (2012) described a lack of relation between the loss of striatal dopaminergic function, assessed by positron emission tomography (PET), and CSF α-synuclein levels, in asymptomatic carriers of mutations in the *LRRK2* gene. CSF neurosin (a protease that degrades α-synuclein) levels have been found to be decreased (Wennström et al., 2013).

Lower baseline CSF α-synuclein levels in the DATATOP study predicted a better preservation of cognitive function in early PD patients with up to 8 years of follow-up (Stewart et al., 2014).

The results of the studies reported on CSF α-synuclein levels in PD are summarized in Table 5. The majority of recent studies have shown decreased CSF α-synuclein levels both in PD and in other synucleopathies. Therefore, this should be a useful marker to distinguish this disease from controls, but not to distinguish among synucleopathies.

**Table 5:** **Results of studies on CSF alpha-synuclein and phosphotau levels in PD, other parkinsonian syndromes and controls**.

**References**	**Cases/Controls**	**Main findings**
Borghi et al., 2000	12 PD, 10 controls	Identification of a 19 kDa band that corresponds to monomeric α-synuclein (similar levels in PD and controls)
Woulfe et al., 2002	5 PD, 4 controls	Similar anti-α-synuclein antibodies in PD and controls
Tokuda et al., 2006	33 PD, 38 controls (9 healthy and 29 with OND)	CSF α-synuclein levels significantly lower in PD than in controls (*p* < 0.0001)
Ohrfelt et al., 2009	66 AD, 15 PD, 15 DLBD, 55 controls	CSF α-synuclein AD > Controls = DLBD = PD
Hong et al., 2010	117 PD, 132 controls, 50 AD	CSF α-synuclein PD < Controls = AD (after correcting for hemoglobin levels)
Tokuda et al., 2010	32 PD, 28 controls (12 healthy and 16 with OND)	CSF α-synuclein oligomers and oligomers/total-α-synuclein ratio in CSF higher in PD group (*p* < 0.0001)
Tokuda et al., 2010	25 PD, 18 PSP, 35 AD, 43 controls	CSF α-synuclein PD > PSP = Controls > AD
Parnetti et al., 2011	38 PD, 32 DLBD, 48 AD, 31 FTD, 32 controls with other neurological diseases (*n* = 32)	CSF α-synuclein Controls > PD > DLBD = AD = FTD
Mollenhauer et al., 2011	Cross-sectional cohort: 51 PD, 29 MSA, 55 DLBD, 62 AD, and 72 neurological controls	CSF α-synuclein PD < DLBD < MSA < controls < AD
Kang et al., 2013	Validation cohort: 275 PD, 15 MSA, 55 66 DLBD, 8 PSP, 22 NPH, and 23 neurological controls	CSF α-synuclein MSA < DLBD = PD < NPH = PSP < controls
Park et al., 2011	23 PD, 29 neurological controls	CSF α-synuclein oligomer significantly higher in PD than in neurological controls
Kang et al., 2013	63 PD, 39 controls	Slightly, but significantly, lower CSF levels of α-synuclein in PD compared with healthy controls
		Lower levels of CSF α-synuclein associated with increased motor severity
Hall et al., 2012	90 PDND, 33 PDD, 70 DLBD, 48 AD, 45 PSP, 48 MSA, 12 CBD, 107 controls	CSFα-synuclein AD > PSP = Controls > PDD = DLBD = MSA = CBD = PDND
Tateno et al., 2012	9 AD, 6 DLBD, 11 PD, 11 MSA, 11 neurological controls	CSFα-synuclein levels in AD higher than in controls (*P* < 0.05), and significantly lower in PD (*P* < 0.001), DLBD (*P* < 0.01), and MSA (*P* < 0.05) when compared with AD
Wang et al., 2012	Discovery series: 93 PD, 26 AD, 78 controls, 33 PSP, 16 MSA	CSF Phosphorylated α-synuclein (PS-129) PD > Controls > AD > MSA = PSP
	Replication series: 116 PD, 50 AD, 126 controls, 27 PSP, 25 MSA	CSFα-synuclein MSA < PD < PSP > AD = Controls
		CSF PS-199/α-synuclein ratio MSA > PK > AD > PSP = Controls
Aerts et al., 2012	58 PD, 47 MSA, 3 DLBD, 22 Vascular Parkinsonsim, 10 PSP, 2 CBD, 57 controls	CSFα-synuclein did not differ significantly among the study groups
Foulds et al., 2012	13 PDND, 10 PD with cognitive impairment, 16 PDD, 17 DLBD, 12 PSP, 8 MSA, 20 controls (ventricular CSF obtained post-mortem)	CSF total α-synuclein, oligomeric α-synuclein and phosphorylated α-synuclein similar in PDND, PDCI, PDD, DLBD, PSP, MSA, and control groups
		CSF oligomeric phosphorylated α-synuclein significantly higher in MSA (*p* < 0.001) when compared with the other study groups
Shi et al., 2012	8 symptomatic and 18 asymptomatic carriers of the G2019 mutation in the *LRRK2* gene	Lack of correlation between PET scan evidence of loss of striatal dopaminergic and CSF α-synuclein levels
Mollenhauer et al., 2013	78 PD (drug naive), 48 controls	CSF α-synuclein lower in PD than in controls
Wennström et al., 2013	52 controls, 46 AD,38 PDND, 22 PDD, 33 DLBD	AD > controls > DLBD > PD > PDD
Parnetti et al., 2014a	71 PD (8 of 44 carriers of a mutation in the beta-glucocerebrosidase gene (*GBA1*) 45 controls with other neurological diseases	CSF α-synuclein lower and oligomeric/total α-synuclein ratio higher in PD than in controls
Parnetti et al., 2014b	44 PD and 25 controls with other neurological diseases	CSF total α-synuclein lower and oligomeric α-synuclein higher in PD than in controls. No relation with prognosis and cognitive impairment
van Dijk et al., 2014	53 PD, 50 controls	CSF α-synuclein levels reduced in patients with PD, but not correlated with measures of disease severity, and striatal dopaminergic deficit assessed with neuroimaging
Mondello et al., 2014	22 controls, 52 PD, 34 MSA, 32 PSP, 12 CBD	CSF α-synuclein MSA < PD < PSP < CBD < Controls
Stewart et al., 2014	304 early PD patients enrolled in the DATATOP study. Longitudinal follow-up	CSF α-synuclein showed a longitudinal decrease over follow-up period
		CSF α-synuclein was not correlated with the rate of clinical progression of the motor symptoms
		Lower basal levels of CSF α-synuclein were associated with better preservation of cognitive function

AD, Alzheimer's disease; PD, Parkinson's disease; FTD, frontotemporal dementia; PDND, PD non-demented; PD, PD demented; OND, Other neurological diseases; DLBD, diffuse Lewy body disease; PSP, progressive supranuclear palsy; CBD, corticobasal degeneration; MSA, multiple system atrophy; NPH, normal pressure hydrocephalus.

### Amyloid-beta

Amyloid beta (Aβ) are a group of different lengths peptides resulting from the enzymatic cleavage of the amyloid precursor protein (APP). The most common is the 42 amino-acid long Aβ42. These peptides have a differential trend toward aggregation (specially Aβ1-42) to form amyloid plaques, one of the pathological hallmarks of AD and DLBD. The increased risk for developing cognitive impairment and dementia of PD patients in comparison with the general population makes it reasonable to link AD markers such as Aβ42 to PDD. Several studies have shown similar (Holmberg et al., 2003; Mollenhauer et al., 2006; Ohrfelt et al., 2009; Přikrylová Vranová et al., 2010; Aerts et al., 2011; Parnetti et al., 2011; van Dijk et al., 2013a) or decreased (Sjögren et al., 2002; Compta et al., 2009b; Mollenhauer et al., 2011; Shi et al., 2011; Kang et al., 2013; Nutu et al., 2013a; Vranová et al., 2014) CSF Aβ1-42 (Aβ1-42) in PD patients, with the exception of one study which reports increased levels (Parnetti et al., 2014b). Other found decreased CSF Aβ-1-42 (Mollenhauer et al., 2006; Compta et al., 2009b; Alves et al., 2010; Montine et al., 2010; Siderowf et al., 2010) and Aβ1-40 (Alves et al., 2010) and Aβ1-38 (Alves et al., 2010) only in PDD patients or in PD patients with memory impairment.

Baseline CSF Aβ levels in the DATATOP study, were negatively correlated with disease progression assessed with UPDRS (Zhang et al., 2013). Baseline CSF levels of Aβ1-42 in two studies (Siderowf et al., 2010; Parnetti et al., 2014b); and the combination of lower baseline CSF Aβ, worse verbal learning, semantic fluency and visuoperceptual scores, and thinner superior-frontal/anterior cingulated in precentral regions by 3T-brain-Magnetic Resonance Imaging in another (Compta et al., 2013) have been associated with further cognitive decline in PD patients.

CSF Aβ1-42 levels have been reported as decreased (Parnetti et al., 2008; Andersson et al., 2011; Parnetti et al., 2011) or similar (Ohrfelt et al., 2009; Nutu et al., 2013a) in DLBD than in PDD and PD patients, decreased in MSA (Holmberg et al., 2003; Shi et al., 2011), and decreased in DLBD in comparison with PD, PDD (Hall et al., 2012; Vranová et al., 2014), PSP, MSA, and CBD (Hall et al., 2012).

Alves et al. (2013) reported that patients with PD with the postural instability-gait disorders (PIGD) phenotype had significantly reduced CSF Aβ42, Aβ38, Aβ42/40, and Aβ38/40 levels compared with patients with the tremor-dominant phenotype and controls.

Nutu et al. (2013b) described lower CSF levels of Aβ1-15/16 in PD, PDD, PSP, and MSA compared to CBD, AD, and controls.

Beyer et al. (2013) reported a correlation between CSF levels of Aβ38, Aβ40, and Aβ42, and ventricular size in 73 non-demented PD patients and 18 PD patients with mild cognitive impairment.

The results of the studies reported on CSF Aβ levels in PD are summarized in Table 6. Many of these studies suggest the potential usefulness of CSF Aβ1-42 levels to predict cognitive impairment in PD patients.

**Table 6:** **Results of studies on CSF amiloyd beta (Aβ) levels in PD, other parkinsonian syndromes and controls**.

**References**	**Cases/Controls**	**Main findings**
Sjögren et al., 2002	19 AD, 14 FTD, 11 ALS, 15 PD, 17 controls	CSF Aβ42 markedly decreased in AD = ALS < FTD < PD < controls
Holmberg et al., 2003	36 MSA, 48 PD, 15 PSP, 32 controls	CSF Aβ42 MSA < PSP = controls = PD
Mollenhauer et al., 2006	73 PDD, 23 PDND, 41 controls (non-demented neurological patients)	CSF Aβ42 lower in the PDD patients compared to PDND patients and controls. This observation was most marked (*p* < 0.05) in a subgroup of patients with PDD carrying the apolipoprotein genotype epsilon3/epsilon3
Parnetti et al., 2008	19 DLBD, 18 PDD, 23 AD, 20 PDND, 20 controls	DLBD showed the lowest mean CSF Aβ42 levels, with a negative association to dementia duration. PDD patients had mean CSF Aβ42 similar to those seen in PD patients
Ohrfelt et al., 2009	66 AD patients, 15 PD patients, 15 patients with dementia with Lewy bodies (DLBD) and 55 cognitively normal controls	CSF Aβ42 AD < DLBD < PD = Controls
Compta et al., 2009b	20 PDND, 20 PDD, 30 controls patients	CSF Aβ42 ranged from high (controls) to intermediate (PDND) and low (PDD) levels (*P* < 0.001). In all PD and PDD patients, in PDND, CSF Aβ42 was related with phonetic fluency
Alves et al., 2010	109 PDND, 36 controls, 20 mild AD	CSF Aβ42 (19%; *p* = 0.009), Aβ40 (15.5%; *p* = 0.008), and Aβ38 (23%; *p* = 0.004) significantly decreased in PD compared with controls
		CSF Aβ42 reductions in PD less marked than in AD (53%; *p* = 0.002)
		Associations between CSF levels of Aβ42 (β = 0.205; *p* = 0.019), Aβ40 (β = 0.378; *p* < 0.001), and Aβ38 (β = 0.288; *p* = 0.001) and memory impairment, but not executive-attentional or visuospatial dysfunction
Montine et al., 2010	150 controls (115 >50 years; 24 amnestic Mild Cognitive Impairment (aMCI), 49 AD, 49 PD, 11 PDD 62 PD-CIND (cognitive imparment non-demented)	CSF Aβ42 levels reduced in AD (*p* < 0.001), PD-CIND (*P* < 0.05), and PDD (*P* < 0.01), and similar to those of controls in PD
Přikrylová Vranová et al., 2010	32 PD, 30 controls	CSF Aβ1-42 similar in PD and controls
Siderowf et al., 2010	45 PD, longitudinal follow-up at least 1 year	Lower baseline CSF Aβ1-42 associated with more rapid cognitive decline
		Subjects with CSF Aβ1-42 levels =192 pg/mL declined an average of 5.85 (95% confidence interval 2.11–9.58, *p* = 0.002) points per year more rapidly on the DRS-2 than subjects above that cutoff, after adjustment for age, disease duration, and baseline cognitive status
Aerts et al., 2011	21 PSP, 12 CBD, 28 PD, 49 controls	CSF Aβ1-42 similar in CBD, PSP, PD, and controls
Parnetti et al., 2011	38 PD, 32 DLBD, 48 AD, 31 FTD, 32 controls with other neurological diseases	CSF Aβ1-42 controls = PD > DLBD = AD = FTD
Shi et al., 2011	137 controls, 126 PD, 50 AD and 32 MSA	CSF Aβ1-42 controls = PD = _MSA > AD
Mollenhauer et al., 2011	Validation cohort: 275 PD, 15 MSA, 55 66 DLBD, 8 PSP, 22 NPH, and 23 neurological controls	CSF Aβ1-42 DLBD < MSA = NPH = PD < controls < PSP
Andersson et al., 2011	47 DLBD, 17 PDD	Aβ42 lower in DLBD than in PDD
Kang et al., 2013	63 PD, 39 controls	Slightly, but significantly, lower levels of Aβ1-42 in PD compared with controls
Hall et al., 2012	90 PDND, 33 PDD, 70 DLBD, 48 AD, 45 PSP, 48 MSA, 12 CBD, 107 controls	CSF Aβ1-42 AD < DLBD = PDD = PSP = MSA = CBD = PDND = Controls
Přikrylová Vranová et al., 2012	48 PD (17 early-onset PD, 15 tremor-dominant, 16 non-tremor-dominant), 19 neurological controls, 18 AD	CSF Aβ42 lower in AD than in the other groups, and lower in non-tremor-dominant PD compared with controls
Jellinger, 2012	12 PD (6 tremor-dominant PD and 6 non-tremor-dominant PD), 27 AD, 17 controls	CSF Aβ42 lower in tremor-dominant PD than in non-tremor-dominant PD and AD, and lower in these three groups than in controls
van Dijk et al., 2013a	52 PD, 50 controls	CSF Aβ42 similar in PD and controls
Zhang et al., 2013	403 early stage PD patients enrolled in the DATATOP study	CSF baseline levels of Aβ42 weakly but negatively correlated with baseline Unified Parkinson Disease Rating Scale total scores
Beyer et al., 2013	73 PDND, 18 PD with mild cognitive impairment	Association between CSF Aβ38, Aβ40, and Aβ42 with the radial distance of the occipital and frontal horns of the lateral ventricles in PDND. Negative association between CSF Aβ38 and Aβ42 with enlargement in occipital and frontal horns of the lateral ventricles in the pooled sample, and with enlargemente of the occipital horns in PD with mild cognitive impairment
Nutu et al., 2013a	43 PDND, 33 PDD, 51 DLBD, 48 AD, 107 controls	CSF Aβ1-40 AD < DLDB < PDD < PDND = controls
		CSF Aβ1-42 PDD = DLBD = PDND < controls = AD
		CSF Aβ1-40/Aβ1-42 ratio AD < DLDB < PDD = controls = PD
Compta et al., 2013	27 PDND, longitudinal following (11 developed dementia)	Lower CSF amyloid-β predicted development of dementia together with worse verbal learning, semantic fluency and visuoperceptual scores, and thinner superior-frontal/anterior cingulate and precentral regions
Alves et al., 2013	99 PD *de novo* (39 with postural instability/gait disorders –PIGD—and 60 tremor-dominant—TD), 46 controls	CSF Aβ42, Aβ38, Aβ42/40, and Aβ38/40 levels significantly reduced in PIGD phenotype compared with TD phenotype and with controls (TD similar to controls)
Nutu et al., 2013b	90 PDND, 32 PDD, 68 DLBD, 48 AD, 45 PSP, 46 MSA, 12 CBD, 107 controls	Significantly lower levels of Aβ1-15/16 were detected in PD, PDD, PSP, and MSA compared to other neurodegenerative diseases and controls
Parnetti et al., 2014b	44 PD and 25 controls with other neurological diseases	CSF Aβ42 lower in PD than in controls. This value was related with cognitive impairment
Vranová et al., 2014	27 PDND, 14 PDD, 14 DLBD, 17 AD 24 controls	CSF Aβ42 PDND > PDD > DLBD >AD > controls

AD, Alzheimer's disease; PD, Parkinson's disease; ALS, amyotrophic lateral sclerosis; FTD, frontotemporal dementia; PDND, PD non-demented; PD, PD demented; DLBD, diffuse Lewy body disease; PSP, progressive supranuclear palsy; CBD, corticobasal degeneration; MSA, multiple system atrophy; aMCI, Amnestic Mild Cognitive Impairment; PD-CIND, PD with cognitive imparment non-demented; NPH, normal pressure hydrocephalus; PIGD, Postural instability and gait disorder; TD, tremor-dominant.

### Neurofilament proteins

Abnormal accumulation in the cytoplasm of neurofilaments (NF), members of the cytoskeleton proteins expressed by neurons, have been detected in neurodegenerative diseases including AD, MSA, DLBD, and PD. CSF levels of neurofilament light (NFL) proteins have been found normal in PD patients (Constantinescu et al., 2010; Hall et al., 2012), and increased in patients with PSP (Holmberg et al., 1998; Constantinescu et al., 2010; Hall et al., 2012), MSA (Holmberg et al., 1998; Constantinescu et al., 2010; Hall et al., 2012), CBD (Constantinescu et al., 2010; Hall et al., 2012), and PDD (Hall et al., 2012).

CSF neuronal thread protein (NTP) levels have been found increased when compared with controls and decreased when compared with AD patients in one study (de la Monte et al., 1992), and similar to those of controls in another (Yamada et al., 1993). CSF annexine V has been found to be decreased in PD (Vermes et al., 1999). Glial fibrilar acidic protein (GFAP) has been found to be normal in the CSF of PD, MSA, PSP, and CBD patients (Constantinescu et al., 2010). CSF levels of the glial activation marker YKL-40 have been found to be decreased in PD, MS, PSP, and CBD (Olsson et al., 2013).

### Other proteins

Defects in the gene encoding DJ-1 protein cause an autosomal recessive early-onset PD, PARK7 (Alonso-Navarro et al., 2014). This protein is also a marker of oxidative stress. CSF levels of DJ-1 protein have been found to be increased in PD in 2 studies (Waragai et al., 2006; Herbert et al., 2014) and decreased in another 2 (Shi et al., 2011; Hong et al., 2010). One of these studies described decreased CSF DJ-1 in MSA as well (Shi et al., 2011), and other increased DJ-1 in MSA compared with PD and with controls (Herbert et al., 2014). Shi et al. (2012) described a lack of relation between the loss of striatal dopaminergic function and CSF DJ-1 levels in asymptomatic carriers of mutations in the *LRRK2* gene (PARK8). The results on DJ-1 are, therefore, inconsistent and should not be considered as a marker of PD.

Defects in the gene encoding ubiquitin carboxy-terminal hydrolase 1 (UCH-L1) cause familial PD, PARK5. A recent study found decreased CSF UCH-L1 levels in PD, MSA, and PSP compared with controls (Mondello et al., 2014).

Among proteins related with apoptosis, Bcl-2 protein has not been detected in the CSF of PD patients (Mogi et al., 1996b; Mogi and Nagatsu, 1999). CSF levels of clusterin have been reported to be increased (Přikrylová Vranová et al., 2010; Vranová et al., 2014) or normal (van Dijk et al., 2013a), tissue transglutaminase (Vermes et al., 2004) increased in PD, and cystatin C normal in PD (Přikrylová Vranová et al., 2010; Yamamoto-Watanabe et al., 2010) and decreased in MSA (Yamamoto-Watanabe et al., 2010).

Studies measuring CSF levels of lysosomal hydrolases (involved in the α-Syn degradation) found decreased (Balducci et al., 2007), normal (van Dijk et al., 2013b), or increased (Parnetti et al., 2014a) β-hexosaminidase, increased cathepsin E (van Dijk et al., 2013b), decreased α-mannosidase (Balducci et al., 2007), decreased (Balducci et al., 2007) or normal β-mannosidase (Mollenhauer et al., 2011; van Dijk et al., 2013b), decreased α-fucosidase (van Dijk et al., 2013b), β-glucocerebrosidase decreased (Balducci et al., 2007; Parnetti et al., 2014a) or normal (van Dijk et al., 2013b), β-galactosidase increased (van Dijk et al., 2013b) or normal (Balducci et al., 2007; Parnetti et al., 2014a), and cathepsin D normal (van Dijk et al., 2013b) in PD patients compared with controls.

CSF Prion protein (PrP) (Meyne et al., 2009) and tetranectin (involved in tissue remodeling) (Hong et al., 2010) levels have been found to be decreased, and apolipoprotein A-1 normal (Wang et al., 2010) in PD patients. CSF levels of transthyretin (TTR, a clearance protein produced in the choroid plexus) have been found to be increased in Lewy body diseases, including PD, PDD, and DLBD in relation with controls (Maetzler et al., 2012). CSF levels of the soluble proteoglycan NG2 (sNG2), involved in proliferation, migration, and differentiation of perycites and NG2 cells in the brain, have been found to be similar in PD patients and controls, and decreased in DLBD (Nielsen et al., 2014).

In PD patients there are reports of decreased CSF post-proline cleaving enzyme (Hagihara and Nagatsu, 1987), increased dipeptidyl-aminopeptidase II (Hagihara et al., 1987), normal dipeptidyl-aminopeptidase IV (Hagihara et al., 1987), and normal glutamic oxaloacetic transaminase (GOT) (Steen and Thomas, 1962; Weiss et al., 1975; Qureshi et al., 1995) and glutamic pyruvic transaminase (GPT) (Weiss et al., 1975) levels.

## Other compounds

In patients with PD there have been reports of normal CSF levels of the proteoglycan N-acetyl neuraminic acid (Lipman and Papadopoulos, 1973), and CSF insulin levels (Jiménez-Jiménez et al., 2000) have been found normal in PD patients.

The CSF levels of corticosterone (Pålhagen et al., 2010) and neuroactive steroids such as allopregnanolone (THP) and 5 α-dihydroprogesterone (DHP) (di Michele et al., 2003) have been found to be decreased in PD. Björkhem et al. (2013) reported that 10% of the PD patients were found to have increased CSF levels of 24S-hydroxycholesterol, and that there was a significant correlation between this value and duration of the disease. Lee et al. (2008) described a significant increase in the CSF levels of the polyunsaturated fatty acid eicosapentanoic acid (EPA) in patients with PD and MSA.

Paik et al. (2010) measured several polyamines in the CSF of patients with PD, MSA and controls. These substances are important for cell growth, and act as important modulators of a variety of ion channels, including glutamate NMDA and AMPA receptors. CSF total polyamine, N^1^acetyl-cadaverine, and cadaverine levels were increased both in PD and MSA, but PD patients showed higher CSF putrescine and lower CSF spermidine levels than MSA and controls, and MSA patients showed lower CSF N^1^acetylputrescine than PD and controls. CSF N^8^-acetylspermidine levels were higher in PD patients than in controls, and in MSA than in PD patients and controls.

## Conclusions

- The majority of classical biochemical studies on neurotransmitter and related substances have described decreased CSF HVA, and normal NE, MHPG, ACh, AChE, glutamate, aspartate, and glycine levels in patients with PD. Results on CSF GABA and 5-HIAA levels are controversial. Many of these classical studies included patients with different types of Parkinsonism and had a limited number of patients and controls.
- Studies on the possible value of endogenous neurotoxins, oxidative stress markers, inflammatory and immunological markers, and growth and neurotrophic factors as biological markers of PD should be considered as inconclusive. The most consistent finding related with these issues is the possible role of CSF urate on the progression of the disease (Ascherio et al., 2009).
- Data regarding the role of CSF total *tau* and phospho*tau* as biological markers for PD are inconsistent. The most interesting findings are the possible relations of these markers with the progression of the disease (Zhang et al., 2013), and with the preservation of cognitive function in PD patients (Stewart et al., 2014).
- CSF α-synuclein levels have been found to be decreased in most, but not all, studies in PD patients compared with controls. This marker should be useful for the differential diagnosis between synucleopathies and other parkinsonian syndromes, but its usefulness to differentiate among synucleopathies (PD, PDD, DLBD, and MSA), remains to be elucidated.
- CSF Aβ1-42 levels could be considered as a useful marker of the presence of further cognitive decline in PD patients.
- CSF NFL protein levels should be useful for the differential diagnosis of PSP, MSA, CBD, and PDD from PD, but not to discriminate between PD and healthy controls.

## Future approaches

While possible biomarkers for PD in classical studies have been hypothesis-driven, attempts to develop effective procedures for the differential diagnosis of PD in its early stages have led to the performance of CSF multianalyte methods including systematic measurements of patterns of variation in proteins (proteomics) or small molecules (metabolomics). These methods have led to the identification of possible unexpected biomarkers of diseases involved in neurodegenerative processes. However, the results of these types of studies, which are briefly described below, are not clearly established and await replication.

Guo et al. (2009), in a proteomic analysis of the CSF of PD patients and controls, found significantly higher CSF levels of apolipoprotein E, autotoxin, and some SOD1 isoforms, and lower levels of complement C_4_ when compared with controls, while Pigment epithelium-derived factor (PEDF or serpin F1) was not significantly increased, and complement C_3_ and haptoglobin were similar in PD patients and controls.

Zhang et al. (2008) performed a proteomics-discovered multianalyte profile (MAP) in CSF on 95 control subjects, 48 patients with probable AD, and 40 patients with probable PD, and concluded that the optimal MAP leading to the correct diagnosis was composed of the following proteins in order of contribution: tau, BDNF, IL-8, Aβ42, β2-microglobulin, vitamin D binding protein, apoA2, and apoE.

Maarouf et al. (2012) analyzed ventricular CSF from PD and controls obtained in the immediate post-mortem period using a two-dimensional difference gel electrophoresis (2D-DIGE) coupled with mass spectrophotometry protein identification, and found differences between the 2 groups in 6 molecules: fibrinogen, transthyretin, apoE, clusterin, apoA1, and glutathione-S-transferase-Pi (GSTP).

Trupp et al. (2014) reported a generally lower level of metabolites in PD as compared to controls, with a specific decrease in 3-hydroxyisovaleric acid, tryptophan, and creatinine, a significant decrease in the levels of Aβ-38 and Aβ-42, and an increase in soluble amyloid peptide precursor α (APPα) in CSF of patients.

Ideally, future studies should fulfill the following conditions: (a) a multicenter and prospective design; (b) inclusion of patients diagnosed with PD and other types of parkinsonism according to standardized criteria; (c) measurement of multiple potential biological markers in the CSF; (d) a very long-term follow-up period (till death as end-point), with assessment of both clinical features and serial determinations of the biological markers; and (e) final neuropathological confirmation by examination of the brains of patients at death (this is lacking in most of the studies published).

### Conflict of interest statement

The authors declare that the research was conducted in the absence of any commercial or financial relationships that could be construed as a potential conflict of interest.
